# Improving of hydrolases biosythesis by solid-state fermentation of *Penicillium camemberti* on rapeseed cake

**DOI:** 10.1038/s41598-018-28412-y

**Published:** 2018-07-05

**Authors:** Filip Boratyński, Ewa Szczepańska, Aleksandra Grudniewska, Radosław Gniłka, Teresa Olejniczak

**Affiliations:** Department of Chemistry, Wroclaw University of Environmental and Life Sciences, Wrocław, 50-375 Poland

## Abstract

The study show usefulness of rapeseed cake, rich in fats and proteins byproduct generated after oil production, which may be used as a microbial medium for lipase and protease biosynthesis. Of 26 different filamentous fungi screened by solid-state fermentation, *Penicillium camemberti* AM83 was found to abundantly produce lipase and protease. Various process parameters were then optimized to maximize lipase and protease secretion, including carbon and nitrogen source, C/N ratio, metal ions, temperature, moisture content, initial pH, and inoculum size. Lipase production increased approximately 11.2-fold in solid-state cultures on rapeseed cake supplemented with lactose and calcium chloride, alkalinized to pH 8, hydrated to 80%, and inoculated with 1.2 × 10^6^ spores/mL. Similarly, protease production increased approximately 8.4-fold in optimized cultures inoculated with 3.2 × 10^8^ spores/mL, and grown on rapeseed cake with lactose and ammonium sulfate at pH 9 and moisture content 60%. The results highlight the potential economic value of solid-state fermentation on rapeseed cake to produce industrial hydrolases.

## Introduction

Solid-state fermentation is defined as a technique for growing microorganisms on moistened solid substrates. It has emerged as a technology for the production of fuel, food, pharmaceutical products and industrial chemicals. Solid-state fermentation is applied in bioprocesses such as bioremediation, bioleaching, biobeneficiation, biopulping^1^.

In recent years, particular attention has been paid to reducing or exploiting food waste. Among them are oilseed cakes, wheat bran, beet, potato and fruit pulps. These residues are abundant in carbohydrates, protein, fats, and cellulose, but low in water, and are thus excellent media for filamentous fungi^2^. To metabolize available nutrients, fungi produce various enzymes, principally hydrolases such as lipase^3^. Of note, Castilho *et al*.^4^ found that the capital investment required to produce industrial lipase is 78% lower for solid-state fermentation than for submerged fermentation. Solid-state fermentation is also more efficient and more sustainable^5^. Since growth media account for around 40% of the total cost to produce the enzyme, inexpensive raw materials such as agricultural byproducts are more economical^6^. Therefore, microbial solid-state fermentation on renewable agroindustrial residues is ideal to efficiently and inexpensively produce not only lipases but also other industrial biocatalysts such as proteases, cellulases, and amylases^7^.

Rapeseed, also known as colza, is cultivated primarily in the European Union, China, Canada, India, and Australia, mainly to produce food-grade vegetable oil and animal feed. With increased interest in biofuels in recent years, rapeseed oil has also now become the primary feedstock for biodiesel production. About 70 million tons of rapeseed are produced globally every year, implying that large quantities of residual rapeseed cake are available for further exploitation, especially in Europe, where rapeseed cake is one of the major byproducts of the oleochemical industry^8^. Rapeseed cake constitutes valuable raw material for production industrially important hydrolases, especially lipases and proteases. This is due to the high content of lipids (~13%) and proteins (~40%)^9,10^. Microorganisms to assimilate these nutritional compounds produce particular enzymes.

Lipases constitute one of the most important industrial enzymes, and are used in various industrial and scientific processes that require hydrolysis, esterification, transesterification, and alcoholysis^11–13^. Due to substrate specificity, regio-, and enantioselectivity, lipases are, indeed, widely used to manufacture food, pharmaceuticals, cosmetics, detergents, and paper^14–16^. Proteases are similarly valuable commercially, and are widely used at industrial scale to produce detergent, pharmaceuticals, leather, and silk. Besides, they play substantial role in food industries production processes, especially in cheese, meat, fish, bakery, brewing, fermented food. Proteases improve the taste of the products and facilitate the process of processing^17^. Production of both, lipases and proteases, could be interesting due to the fact that both groups of hydrolases are used in cheese production. Lipases induce cheese ripening and enhance their aroma, while fungal proteases are responsible for coagulation of milk proteins and used in production of cheddar cheese.

The purpose of this study was to find a fungal strain that efficiently produces lipase as well as protease from rapeseed cake, as measured by quantitative enzyme activity assays of crude extracts. Based on an initial screening, *Penicillium camemberti* AM83 was selected from 26 other filamentous fungi. This strain is non-pathogenic, and is widely used to ripen cheese. Process optimization highlighted the impact of culture conditions, carbon, and nitrogen sources on enzyme secretion. Collectively, the data suggest that *Penicillium camemberti* has additional potential value as source of industrial enzymes.

## Materials and Methods

### Microorganisms

The following filamentous fungi strains were used for screening: *Aspergillus* sp. AM31, *Aspergillus candidus* AM386, *Aspergillus glaucus* AM211, *Aspergillus nidulans* AM243, *Aspergillus ochraceus* AM456, *Aspergillus wenthi* AM413, *Botrytis cirenea* AM235, *Fusarium avenaceum* AM11, *Fusarium equiseti*AM15, *Fusarium oxysporum* AM13, *Fusarium oxysporum* AM21, *Fusarium semitectum* AM20, *Fusarium solani* AM203, *Fusarium tricinctum* AM16, *Mucor spinosus* AM398, *Papularia rosea* AM17, *Penicillum camembertii* AM83, *Penicillium chrysogenum* AM112, *Penicillium frequentans* AM351, *Penicillium notatum* AR904, *Penicillium thomi* AM91, *Penicillium vermiculatum* AM30, *Poria placenta* AM38, *Sclerophama pythiophila* AR55, *Spicoria divaricata* AM423, *Syncephalastrum racemosum* AM105. The microorganisms were derived from Department of Chemistry at Wroclaw University of Environmental and Life Sciences (Poland). They were stored at 4 °C on Sabouraud agar slants containing peptone (10 g), glucose (30 g) and agar (15 g) dissolved in water (1 L) at pH 5.5.

### Strain selection by solid-state fermentation

Rapeseed cakes from Oleofarm, Wroclaw, Poland (10 g each) were placed in 300 mL Erlenmeyer flasks, hydrated to 60% moisture, autoclaved for 15 min at 121 °C, inoculated with a dense spore suspension (2.3 × 10^7^ spores/mL) prepared in sterile water from agar slant cultures, and thoroughly mixed. Flasks were then incubated at 25 °C without shaking. To assess lipase and protease production, samples were collected after 2 and 5 days respectively.

### Process optimization

The effect of carbon and nitrogen sources was assessed by supplementing solid-state cultures with 2% (w/w) simple or complex carbon (arabinose, mannose, ribose, soluble starch, lactose, fructose, saccharose, maltose, glucose, xylose, glycogen, olive oil) and organic and inorganic nitrogen (peptone, yeast extract, soy flour, ammonium nitrate, ammonium sulfate, sodium nitrate, ammonium tartrate, and diammonium citrate). The impact of the C/N ratio (C: lactose for lipases and proteases; N: peptone for lipases and ammonium sulfate for proteases) was also evaluated, along with that of 0.2% w/w metal ions (CaCl_2_, NaCl, sodium citrate, MgSO_4_, MnSO_4_, and ZnSO_4_) and surfactant (Triton X-100). Incubation temperature was tested from 20 to 40 °C, while initial pH was assessed at pH 3–5 in 0.1 M citrate buffer, pH 6–8 in phosphate buffer, and pH 9–11 in Tris base buffer. Moisture content was tested at 40%, 50%, 60%, 70%, 80%, and 90%, while inoculum size was examined using inocula with corresponding suspension of spores: 1.2 × 10^6^, 2.3 × 10^7^ and 3.2 × 10^8^ spores/mL.

### Enzyme extraction

Samples of solid-state media (5 g) were vortexed for 5 min at 3,500 rpm in phosphate buffer pH 7.2, centrifuged at 6,700 × *g* for 10 min at room temperature, and assayed for lipase and protease activity. The total protein content in crude extract was estimated by the method of Bradford, using a bovine serum albumine as a standard. In control experiments, which constituted oilseed cakes without microorganism, analogous procedure was conducted.

### Lipase activity assay

Lipase activity was determined in a spectrophotometric assay with *p*-nitrophenyl palmitate (pNPP, Sigma) as a substrate according to the Mahdi *et al*.^18^ with some modifications. The enzyme reaction mixture contained 75 μL of substrate (1 mM) dissolved in isopropanol, 50 μL of crude enzyme filled to 3 mL by 50 mM Tris-HCl buffer (pH 8) and incubated at 37 °C for 10 min. The reaction was interrupted by addition of 1 mL cooled ethanol. The activity was measured at 410 nm. One enzyme unit (U) was defined as an amount of enzyme that released 1 μM *p*-nitrophenol per minute. Lipase activity was calculated using *p*-nitrophenol standard curve. Lipase activity was expressed in units/gram of rapessed cake/mg of protein contained in crude extract.

### Protease activity assay

Protease activity was determined according to the Afifi *et al*.^19^ with some modifications. Protease activity in crude extract was measured in a mixture containing 1 mL of casein solution (0.6% in 0.1 M Tris-HCl buffer, pH 8), 1 mL of phosphate buffer, 0.5 mL of crude enzyme. The reaction was performed for 30 min at 37 °C, and then 5 mL of 0.11 M TCA was added to the mixture to terminate the reaction. Subsequently, mixture was filtered and the optical density was measured at 275 nm. One unit of enzyme was defined as an amount of the enzyme required to liberate 1 μg of tyrosine per minute. Protease activity was expressed in units/gram of rapessed cake/mg of protein contained in crude extract.

### Statistical analysis

Statistical analyses (standard deviation and t-test for unpaired samples) were performed by Statistica (StatSoftPoland, ver. 13.1). The tests were two-tailed, alpha level was equal 0.05. Error bars are the mean ± standard deviation from three independent experiments.

## Results and Discussion

### Strain selection

**Figure 1:**
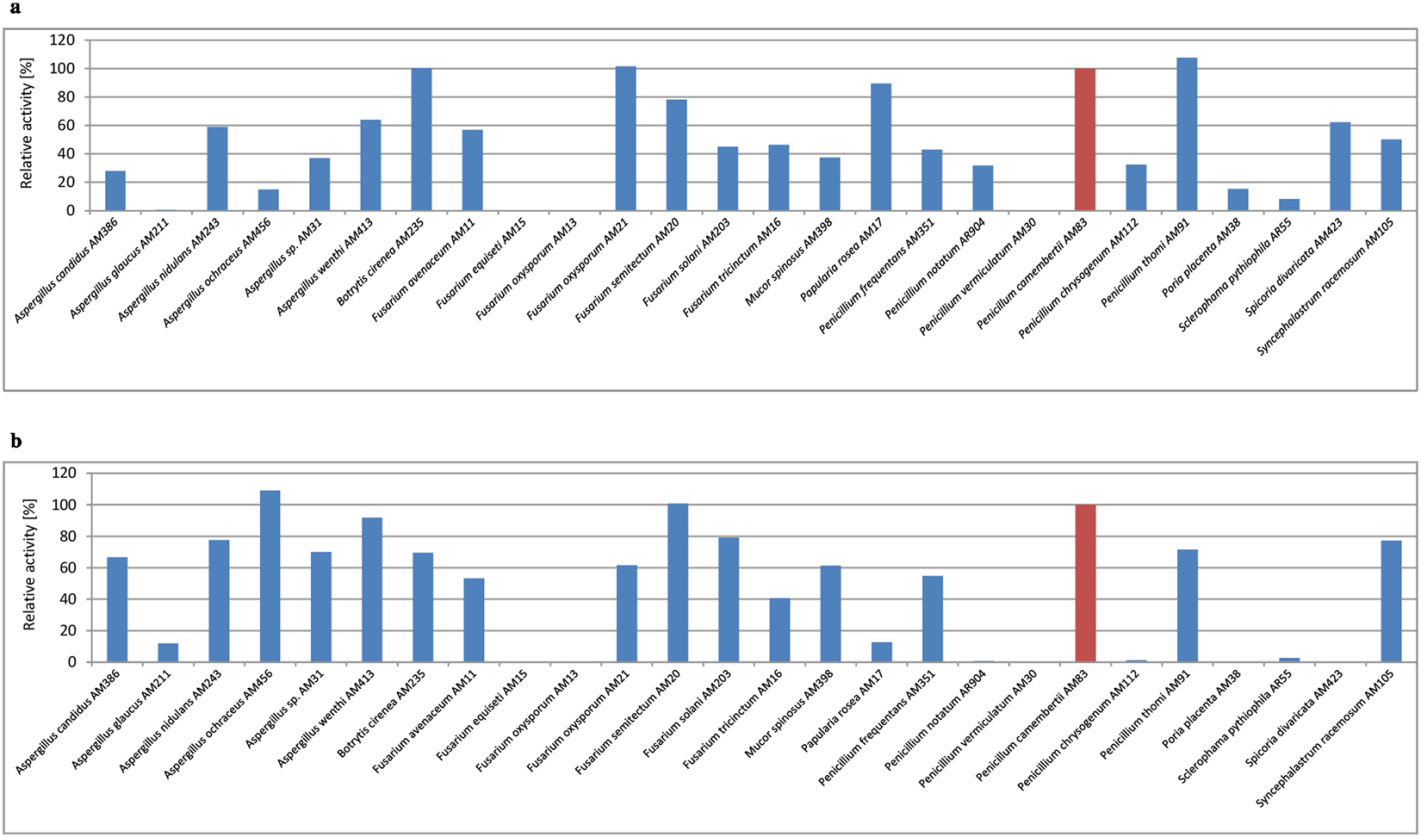
Comparison of lipase (**a**) and protease (**b**) activity determined after 2 and 5 days respectively in solid-state fermentation relative to *P*. *camemberti* AM83 (100%).

In initial experiments, twenty six filamentous fungi, mainly *Aspergillus*, *Fusarium*, *Penicillium* as well as *Botrytis*, *Mucor*, *Papulari*, *Poria*, *Sclerophama*, *Spicoria*, and *Syncephalastrum* were tested in solid-state fermentation on rapeseed cake to identify the most prolific producer of lipase and protease. As can be seen in Fig. 1, lipase secretion was highest in *P*. *camemberti* AM83, *B*. *cirenea* AM235, *F*. *oxysporum* AM21, and *P*. *thomi* AM91, while protease production was highest in *P*. *camemberti* AM83, *F*. *semitectum* AM20, and *A*. *ochraceus* AM456. Notably, efficient producers of lipase were weak producers of protease and vice versa, with the exception of *P*. *camemberti* AM83, which abundantly produced both enzymes. Hence, *P*. *camemberti* AM83 was selected for further optimization since it abundantly produces both lipase and protease. In order to underline the distinctive biosynthesis of the desired hydrolases by *P*. *camemberti* AM83, the hydrolytic activity of all screened fungi in relation to this strain has been represented (Fig. 1).

Various physical and chemical factors are well-known to affect enzyme production from microbial cultures. Accordingly, we found that lipase production increased from 0 to 48 h of culture, but decreased thereafter. In contrast, protease production increased gradually and peaked at 120 h (data not shown). This pattern is probably due to the abundance of lipids in rapeseed cake, which are thus metabolized first (2^nd^ day), while proteins are metabolized later (5^th^ day), presumably after lipids have been depleted.

While solid-state fermentation is widely used to produce enzymes by fungi, we are aware of only one report to date describing lipase production in solid-state (wheat bran) cultures of *P*. *camemberti*^20^. In addition, protease production in this species has not been investigated, although lipase and protease production by solid-state fermentation on various substrates has been reported for other *Penicillium* species. For example, *P*. *simplicissimum*^21,22^, *P*. *verrucosum*^23^, and *Penicillium* sp.^24–26^ have been grown on soybean meal, while *P*. *simplicissimum*^27^, *P*. *brevicompactum*^28^, and *P*. *restrictum*^29–31^ have been cultivated on babassu cake.

### Optimization of culture conditions

#### Carbon source

**Figure 2:**
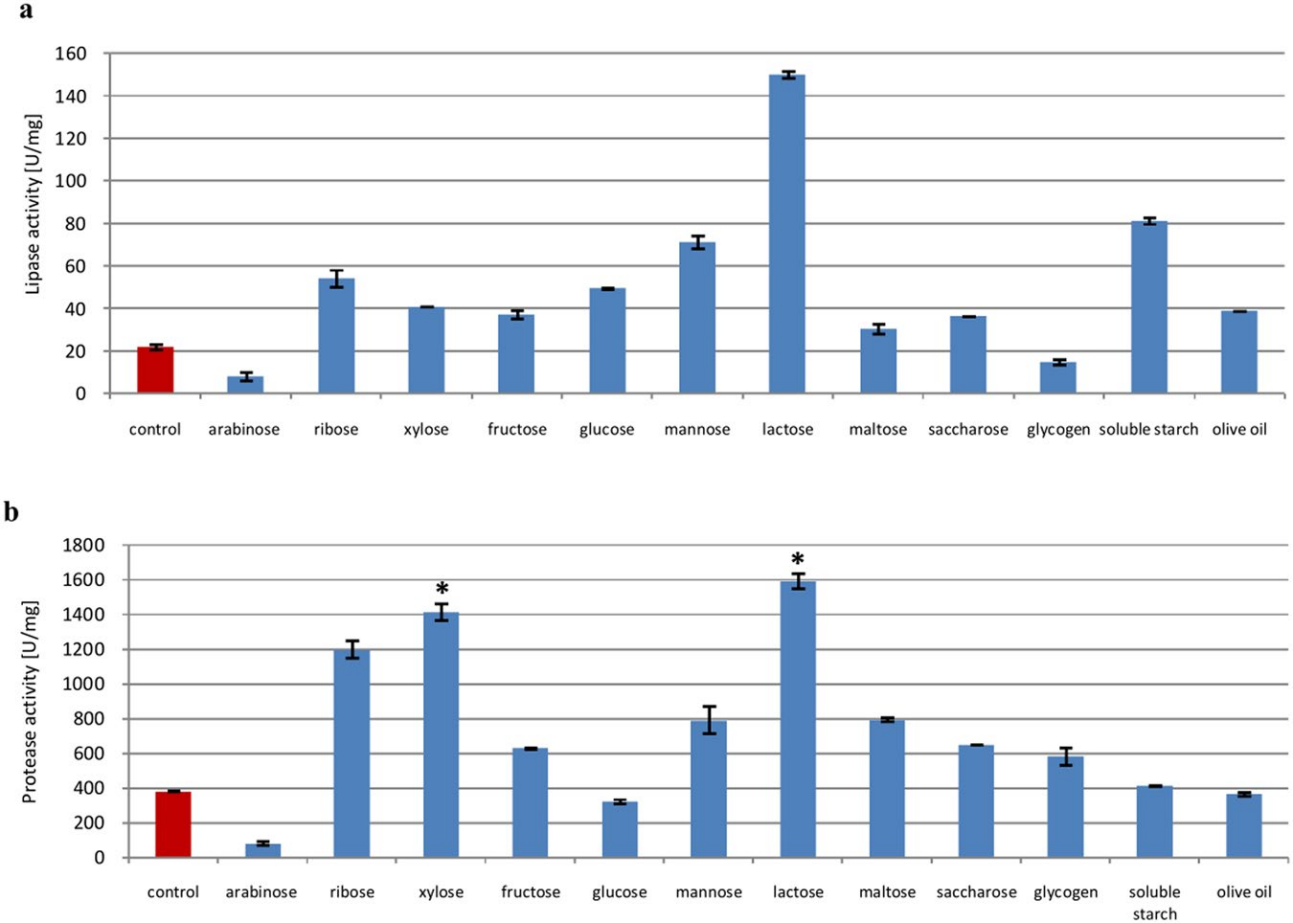
Effect of carbon source on lipase (**a**) and protease (**b**) biosynthesis. Error bars are the mean ± standard deviation from three independent experiments. Statistical significance was *P = 0.008.

Cultures of *P*. *camemberti* AM83 were supplemented with 12 different carbon sources to test effects on lipase and protease biosynthesis. Results show that carbon supplements generally enhanced enzyme secretion (Fig. 2). In particular, all carbon sources except arabinose and glycogen enhanced lipase production, with an increase from 22 U/mg (control) to 31–41 U/mg (1.4–1.9-fold) from supplementation with maltose, saccharose, fructose, olive oil, and xylose, and 49–81 U/mg (2.2–3.7-fold) from supplementation with glucose, ribose, mannose, and soluble starch. Similarly, mannose, maltose, ribose, and xylose boosted protease production from 382 U/mg (control) to 791–1415 U/mg (2.1–3.7-fold), while fructose, saccharose, and glycogen increased protease secretion by 1.5–1.7-fold (583–647 U/mg). Lactose was particularly the most effective carbon source, and increased lipase and protease production 6.8- (150 U/mg) and 4.2-fold (1975 U/mg), respectively.

We found that lactose was the optimal carbon source to produce lipase from *P*. *camemberti*, as was noted by Tan *et al*.^32^. Lactose was also tested in other *Penicillium* species by Rehman^33^ and Bancerz *et al*.^34^, although only Amin and Bhatti^35^ found that it increased lipase production. In other cases, olive oil^20,29–31,33,35^ and soluble starch^29,31^ were found to stimulate lipase production in solid-state cultures. We also noted that lactose increased protease production, although protease biosynthesis increased in *Bacillus cereus*^36^ and *Aspergillus versicolor* CJS-98^37^ after supplementation with maltose.

#### Nitrogen source

**Figure 3:**
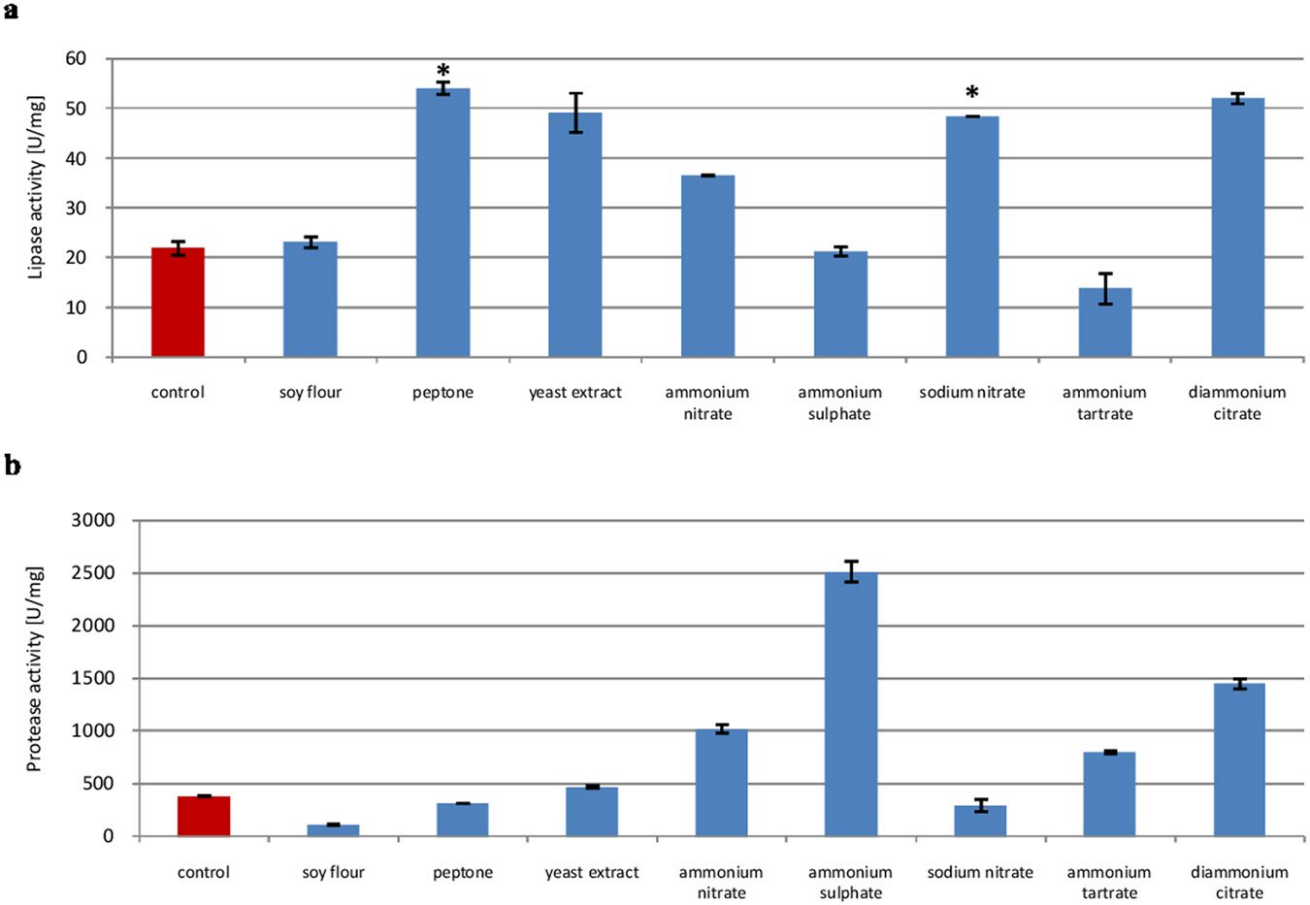
Effects of organic and inorganic nitrogen source on lipase (**a**) and protease (**b**) biosynthesis. Error bars are the mean ± standard deviation from three independent experiments. Statistical significance was *P = 0.001.

Both organic and inorganic nitrogen relieve catabolite repression and induce hydrolase synthesis. Inorganic nitrogen is rapidly consumed by microorganisms, while organic sources provide amino acids and growth factors. Most of the nitrogen sources tested were found to induce lipase production (Fig. 3), while ammonium ions (NH_4_^+^) stimulated protease production. In particular, ammonium sulfate increased protease activity to 2130 U/mg (5.6-fold).

Similarly, various nitrogen sources have been shown to affect lipase production, although effects vary among strains. In *P*. *camemberti*, we found peptone to be most suitable, in line with published data^29,30^. Of note, Rehman^33^ reported that peptone reduced lipase production in *P*. *notatum*. On the other hand, ammonium sulfate induced lipase production in other *Penicillium* strains, as reported by Amin and Bhatti^35^, Malilas *et al*.^20^, and Tan *et al*.^32^. In any case, we found that ammonium sulfate maximized protease production, in contrast to previous studies indicating that peptone or yeast extract was more advantageous^19,30,37,38^.

#### C/N ratio

**Figure 4:**
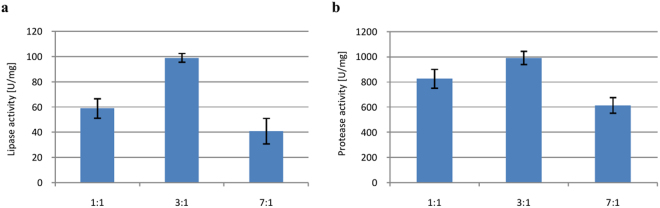
Effects of C/N ratio on lipase (**a**) and protease (**b**) biosynthesis. Error bars are the mean ± standard deviation from three independent experiments. Statistical significance was *P = 0.0017.

As can be seen in Fig. 4, the optimal C/N ratio was 3:1 for both lipase and protease production. We note, however, that while lactose was optimal as carbon source for producing both enzymes, peptone was the most suitable nitrogen source for producing lipase, while ammonium sulfate was optimal for producing protease. The value of C/N ratio had a significant impact on lipase and protease activity as was reported by Rigo *et al*.^24^, Dai and Xia^39^, Palma *et al*.^30^, and Lima^40^.

#### Metal ions and surfactant

**Figure 5:**
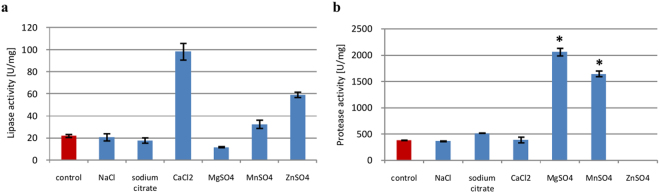
Effects of metal ions on lipase (**a**) and protease (**b**) biosynthesis. Error bars are the mean ± standard deviation from three independent experiments. Statistical significance was *P = 0.001.

Lipases are usually used in processes that require detergents, although such detergents may actually inhibit lipase activity. Indeed, we found that supplementation with 0.2% Triton X-100, a non-ionic surfactant widely used to extract membrane components, inhibited *P*. *camemberti* AM83 lipase and protease activity by 0.9 and 0.5-fold (from 22 to 3 U/mg and from 382 to 195 U/mg). Similarly, metal ions may directly or indirectly impact enzymatic activities. We found that while NaCl did not affect lipase and protease activity, calcium ions stimulated lipase activity approximately 4.0-fold (to 98 U/mg) while magnesium ions increased protease biosynthesis 5.4-fold (to 2059 U/mg). Zinc ions was found to inhibit protease activity, but stimulate lipase activity, while manganese ions stimulated both lipase and protease biosynthesis (Fig. 5).

The observed stimulatory effect of calcium ions on *P*. *camemberti* AM83 lipase was reported for other *Penicillium* species, including *P*. *chrysogenum* 9′^34^, *P*. *candidum*^41^, and *P*. *expansum*^42^. However, Ca^2+^ also inhibited lipase activity in *P*. *citrinum*^43^ and *P*. *camemberti Thom* PG-3^32^. Consistent with Lima *et al*.^44^, we also noted that Zn^2+^ boosted lipase activity, although it inhibited *P*. *chrysogenum* 9′ lipase^34^. However, zinc ions completely inhibited protease activity, while magnesium ions increased it. These results correlate with those of Abidi *et al*.^45^, Afifi *et al*.^19^, Papagianni and Sergelidis^46^, and Zhu *et al*.^47^, who reported similar effects in solid-state cultures of *P*. *italicum* and in submerged cultures of *P*. *chrysogenum*, *P*. *nalgiovense*, and *P*. *chrysogenum* FS010.

#### Incubation temperature

**Figure 6:**
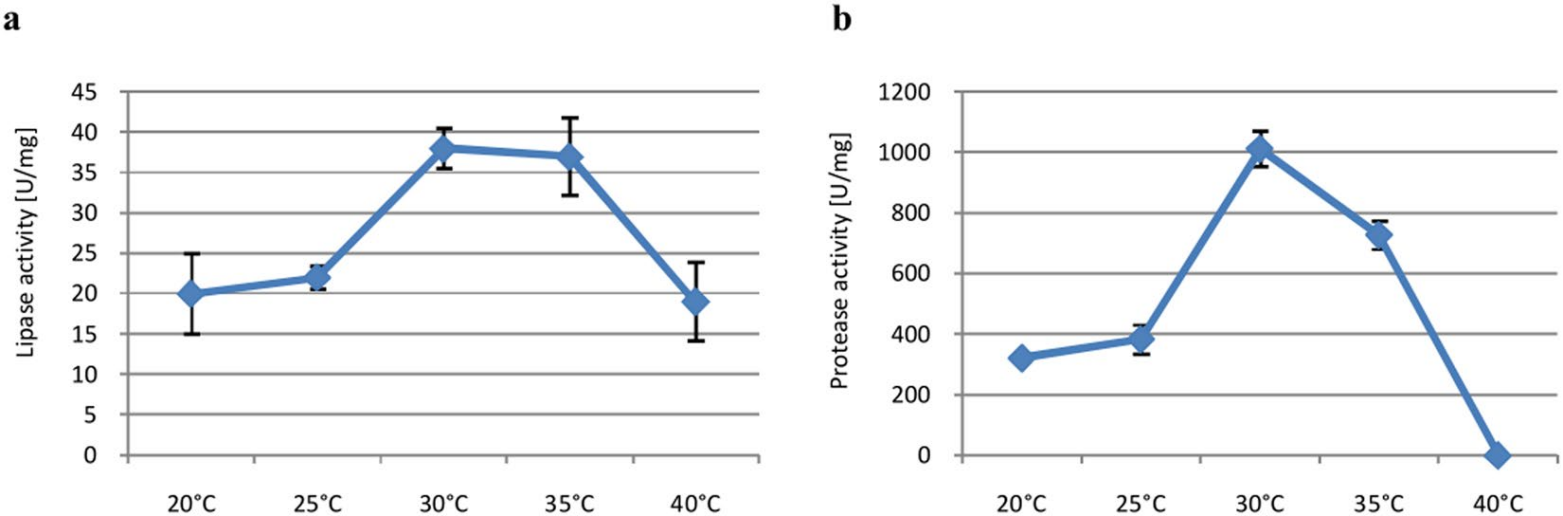
Effects of incubation temperature on lipase (**a**) and protease (**b**) biosynthesis. Error bars are the mean ± standard deviation from three independent experiments.

The optimum temperature for lipase and protease biosynthesis was 30 °C, based on solid-state fermentation tested in the temperature range between 20 °C and 40 °C (Fig. 6).

The optimal incubation temperature for lipase production in *P*. *camemberti* AM83 is similar to that of *P*. *fellutanum* growing on canola cake^35^, *P*. *camemberti* KCCM 11268 growing on wheat bran^20^, and *P*. *simplicissimum* growing on babassu cake^27^. For protease production, the same optimal temperature was observed for *P*. *verrucosum* cultivated on babassu and canola cake^48^ as well as for *P*. *chrysogenum* and *P*. *chrysogenum* IHH_5_^49^ in submerged cultures.

#### Moisture content

**Figure 7:**
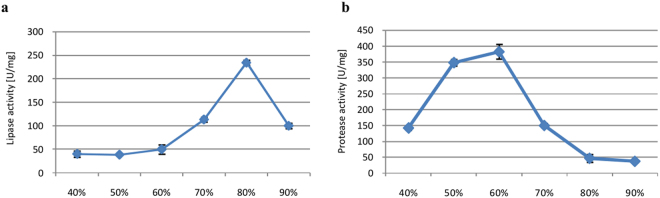
Effects of moisture content on lipase (**a**) and protease (**b**) biosynthesis. Error bars are the mean ± standard deviation from three independent experiments.

On one hand, excessively low moisture content would decrease the solubility of solid media, diminish swelling, and generate higher water tension. On the other hand, excessively high moisture content would reduce porosity, limit oxygen transfer, and increase the risk of contamination. Hence, we investigated lipase and protease production in *P*. *camemberti* AM83 cultured on rapeseed cake with 40 to 90% moisture content (Fig. 7). Lipase and protease activities were highest in media with 80% and 60% water, respectively. Lipase activity was lowest in media with 40–60% moisture, while protease activity sharply decreased in media with 70% moisture and was lowest in media hydrated to 90%.

A level comparable to revealed moisture content was reported by Rigo *et al*.^24^. Similarly, the optimal moisture content for lipase synthesis was estimated by Gutarra^27^ and Silva *et al*.^28^ to be 70% for *P*. *simplicissimum* and *P*. *brevicompactum* in solid-state cultures on babassu cake and castor meal. For protease production, we found that 60%, a level similar to moisture was optimal (55%) reported by Germano *et al*.^26^ for *Penicillium* sp. growing on soybean cake and that (50%) reported by Chutmanop *et al*.^50^ for *Aspergillus oryzae* growing on rice bran.

#### pH

**Figure 8:**
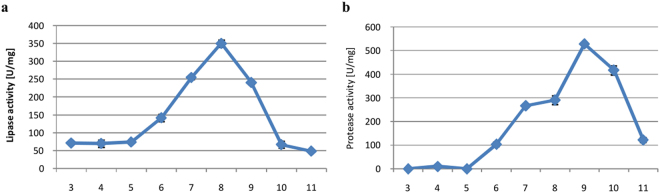
Effects of pH on lipase (**a**) and protease (**b**) biosynthesis. Error bars are the mean ± standard deviation from three independent experiments.

Initial pH is a critical factor in any fermentation process. Accordingly, we found that lipase activity was lowest at pH 3–5 (Fig. 8), but gradually increased with pH, peaking at pH 8 before sharply decreasing at pH 9–11. Similarly, protease production was negligible at pH 3–5, but increased at pH 6–8 before peaking at pH 9. Taken together, the results indicate that lipase and protease were more efficiently produced by *P*. *camemberti* AM83 in alkaline pH.

Most *Penicillium* lipases appear to have suitable pH value between 4 and 7^24,33,35,51–53^. Rarely, the optimum pH is 9, as was observed for lipases from *P*. *simplicissimum* and *P*. *candidum*^41,51^. For *P*. *camemberti* AM83, we found that the optimal initial pH was 8, as was also observed for *P*. *aurantiogriseum*^44^. For protease production, the same optimal initial pH was reported for *P*. *chrysogenum* FS010^47^. We note that for a majority of *Penicillium* proteases, the optimal pH is 8 in submerged cultures^19,54–56^, but is currently unknown for solid-state cultures.

#### Inoculum size

**Figure 9:**
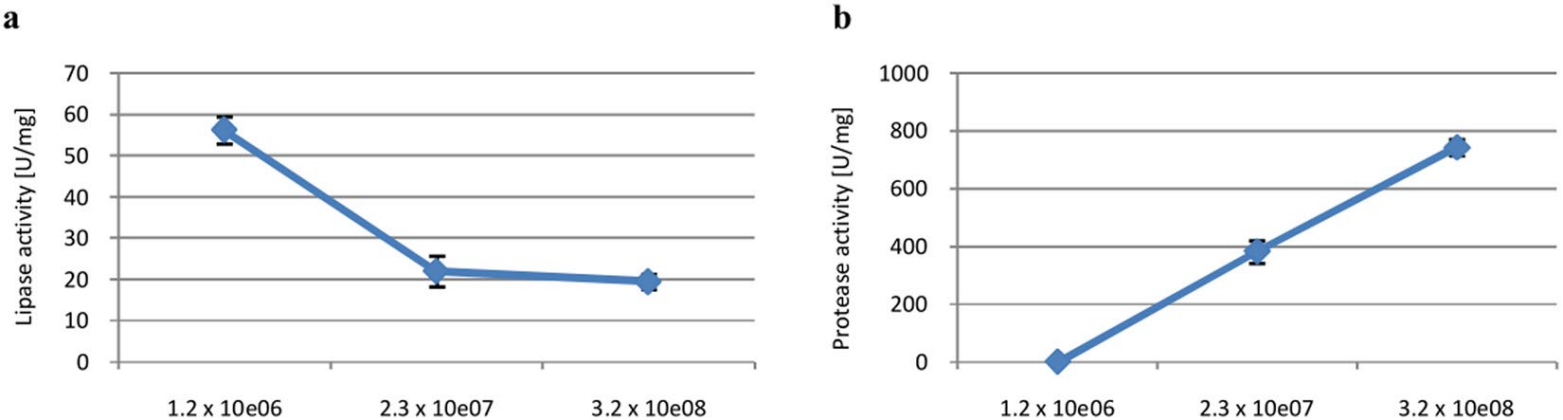
Effects of inoculum size on lipase (**a**) and protease (**b**) biosynthesis. Error bars are the mean ± standard deviation from three independent experiments.

In solid-state fermentation, a suitable inoculum size is required to optimize growth. Too small inocula result in inefficient cell proliferation and production of active metabolites, while too large inocula would rapidly deplete nutrients and prevent sustained growth. As can be seen in Fig. 9, large inocula generally inhibited lipase production, but stimulated protease secretion.

Inocula containing 1.2 × 10^6^ spores/mL and 3.2 × 10^8^ spores/mL were found to maximize lipase and protease production in *P*. *camemberti* AM83. In other *Penicillium* species, the optimal inoculum size was 1 × 10^5^ spores/mL^20^, 1 × 10^7^ spores/mL^51^, 1 × 10^8^ spores/mL^35,57^, and 2 × 10^8^ spores/mL^24^. Of note, Freitas *et al*.^58^ observed that protease production increased with inoculum size.

### Optimized culture conditions

Optimal culture parameters were combined in final fermentation runs. To maximize lipase production, media were hydrated to 80%, alkalinized to initial pH 8, supplemented with 2% w/w lactose and calcium chloride, and inoculated with a suspension of spores 1.2 × 10^6^ spores/mL. An 11.2-fold increase in lipase production was observed as a result (Table 1). Similarly, protease production increased 8.4-fold when rapeseed cakes were supplemented 2% w/w lactose and ammonium sulfate, alkalinized to initial pH 9, hydrated to 60%, and inoculated with a suspension of spores 3.2 × 10^8^ spores/mL.

**Table 1:** Influence of optimized culture conditions on lipase and protease activity.

	Hydrolases activity [U/mg]	
	Lipase	Protease
Basic culture parameters (control)	22	382
Optimal culture parameters	247	3556

Chemical composition of solid media is an essential factor for the production of enzymes. In rapeseed cake high content of carbohydrates and proteins was determined (~30% and ~40% respectively), which makes it attractive medium for microorganism growth. Besides, protein-rich byproduct is an inducer of proteases biosynthesis. The amount of the residual oil is also significant (~15%)^9,59^. The main factor, which can affect on lipases biosynthesis meaningfully is lipids content and fatty acid composition. In rapeseed cake significant majority are unsaturated fatty acids (90–94%). In this agroindustrial side stream quantity of oleic acid prevails (~60%) and it provides ~20% of linoleic acid^59^.

## Conclusion

The scientific significance of the research concerns a number of different aspects. First, production of industrially important enzymes through solid-state fermentation is economically justifiable technique. Second, sustainable development of rapeseed cake, one of the major byproducts of the oleochemical industry in Europe, is undoubtedly important issue from the biorefinery point of view. Third, an initial screening of 26 fungal strains identified *Penicillium camemberti* with GRAS status (Generally Recognised As Safe) used in cheese production as an effective producer of lipase and protease from solid-state rapeseed cake. During the studies, influence of culture conditions on hydrolases biosynthesis was determined. Carbon source and pH proved to be a key factor of lipase production by *P*. *camemberti*. Significant influence of nitrogen and metal ion sources on protease activity was established. However, strong impact revealed also conditions such as temperature of incubation and moisture content. After optimization of culture conditions, lipase activity increased from 22 U/mg to 247 U/mg (11.2-fold), while protease production increased from 382 U/mg to 3556 U/mg (8.4-fold). The results suggest that agroindustrial byproducts such as rapeseed cake has potential as an alternative cost-effective substrate for producing industrial enzymes.
